# Investigation of the psychological health of first‐year high school students with myopia in Guangzhou

**DOI:** 10.1002/brb3.1594

**Published:** 2020-03-08

**Authors:** Qiaoli Li, Jiezheng Yang, Yan He, Ting Wang, Lei Zhong, Ziqian Zhu, Tao Wang, Shiqi Ling

**Affiliations:** ^1^ Department of Ophthalmology The Third Affiliated Hospital Sun Yat‐Sen University Guangzhou China; ^2^ Department of Ophthalmology Nanhai Hospital of Southern Medical University Guangzhou China; ^3^ Department of English Guangzhou Pui Ching Middle School Guangzhou China; ^4^ State Key Laboratory of Ophthalmology Zhongshan Ophthalmic Center Sun Yat‐sen University Guangzhou China

**Keywords:** adolescent, anxiety, depression, myopia

## Abstract

**Purpose:**

The aim of this study was to investigate the differences in anxiety and depression between adolescents with myopia and those with normal vision and to examine the relationship between the level of anxiety and depression and the degree of myopia.

**Methods:**

A total of 1,103 first‐year high school students aged 14–17 years were included in the study. The study group comprised 916 persons with myopia, while the control group comprised 187 persons without refractive error. Volunteers underwent routine eye examinations and completed a set of questionnaires about anxiety and depression. Then, the Self‐Rating Anxiety Scale (SAS) and Self‐Rating Depression Scale (SDS) scores were compared between groups, and the relationships between anxiety and the degree of myopia and between depression and the degree of myopia were analyzed.

**Results:**

There was a significant difference in anxiety rate between the students with normal vision and those with myopia. The SAS scores among students with mild, moderate, and severe myopia were also significantly different. However, compared with the students with normal vision, the rate of depression was not significantly increased in the students with myopia, except in cases of severe myopia. Additionally, the SAS scores correlated closely with the diopters of the participants’ glasses (*r* = 0.43, *p* = .045), while the relationship between SDS scores and the diopters of glasses was not significant (*r* = 0.19, *p* = .325).

**Conclusion:**

There was a correlation between myopia and mental health in adolescent students, especially in terms of anxiety.

## Graphical abstract

There was a correlation between myopia and psychological health in adolescent students, especially in terms of anxiety. And the SAS scores correlated closely with the diopters of the glasses (*p *< .05).

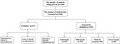

## 1 INTRODUCTION

Myopia, a common eye disease, is one of the leading causes of irreversible blindness worldwide (Morgan et al., 2018). To date, there are approximately 153 million people in the world with visual impairment, and approximately 49% of these impairments are caused by refractive errors. In the past 30 years, the incidence of myopia in the United States has increased from 25% to 41% (Vitale, Sperduto, & Ferris, 2009), and the incidence in some Asian countries, such as Japan, China, and Singapore, has risen even higher, to 70%–90% (Jones & Luensmann, 2012). Myopia, particularly high myopia, is a public health and economic challenge because it is a significant risk factor for other ocular diseases, including glaucoma, diseases of the ocular fundus, and, ultimately, blindness (Morgan, Ohno‐Matsui, & Saw, 2012).

Recently, it has been reported that myopia not only causes anxiety or/and depression but also contributes to a decline in vision‐related quality of life (VR‐QoL) in patients (Ayaki, Torii, Tsubota, & Negishi, 2016). However, Wu et al. found although the VR‐QoL was greatly affected by the deterioration of visual function indices and psychological symptoms, psychological disorders had a greater effect than visual function components on the VR‐QoL. This finding suggested that alleviating psychological anxiety may be more beneficial than preserving visual function in improving the VR‐QoL (Wu, Kong, Gao, & Sun, 2019). Rose et al. examined 112 patients with myopia in the UK and found that the patients’ quality of life was affected by psychological and physical factors (Rose & Tullo, 1998). Psychological disorders, such as depression and anxiety, often develop as comorbidities in cases of chronic and nonchronic somatic illnesses, for example, cardiac infarction, rheumatoid arthritis, and malignancies (Chandarana, Eals, Steingart, Bellamy, & Allen, 1987; Derogatis et al., 1983; Frasure‐Smith & Lesperance, 2008), which have a negative impact on health‐related quality of life (Andersen et al., 2010; Ferrell & Hassey 1997; Skarstein, Aass, Fossa, Skovlund, & Dahl, 2000). It was reported that 22.0%–25.9% of highly myopic patients had possible or probable depression or anxiety disorders, and the presence of those psychiatric disorders was the major factor associated with the low VR‐QoL in highly myopic patients (Yokoi et al., 2014).

The adolescent stage is an important period of personal physical and psychological development. When young people suffer from common disorders, they often experience anxiety and depression, which may develop into adult anxiety or depression disorders (Stapinski et al., 2014). Currently, myopia has reached epidemic levels in young adults in some urban areas in China, especially among students in the highest grades in Guangzhou, where the condition has risen to rates of 80%–90% (Xiang et al., 2013). Currently, there is a significantly higher incidence of pathological intensification of anxiety or depression among younger adolescents with myopia than in those without myopia (Łazarczyk et al., 2016). In addition, some researchers believe that vision correction for patients with myopia using contact lenses or refractive surgery could improve anxiety symptoms (Owsley et al., 2007). Therefore, it is important to determine whether there is a correlation between myopia and mental health in adolescents, as this information might be useful for developing interventions. However, to our knowledge, few studies are available on this topic.

The aims of the present study are to (a) investigate the differences in anxiety and depression between adolescents with myopia and those with normal vision and (b) to examine whether anxiety and depression in adolescent students are associated with the degree of myopia and/or the diopters of the students’ glasses.

## 2 MATERIALS AND METHODS

### 2.1 Participants

From September 1, 2014, to December 1, 2017, 10 high schools in the Yuexiu District of Guangzhou were selected using the cluster sampling method, and 20 grade‐one classes were randomly selected from these schools. The inclusion criteria were as follows: no history of acute or chronic eye surgery or disease, except myopia; no hyperopia or hyperopic astigmatism; no other physical illness or disability; and no recent major trauma. In addition, persons with a mental disorder were excluded if they were currently taking antipsychotics. Participants were interviewed by a licensed psychiatrist to assess the mental health status. As a result, a total of 1,160 students (boys and girls) with a mean age of 15.3 (range, 14.1–17.5) years were selected. This study complies with the Helsinki Declaration, and the entire investigation process obtained the consent of the schools, the participants, their parents and was approved by the Institutional Human Ethics Committee of the Third Affiliated Hospital, Sun Yat‐sen University.

### 2.2 Procedure

#### 2.2.1 Eye examination

All eye examinations were completed and documented by professional ophthalmologists. The participants underwent routine eye examinations, including uncorrected vision, corrected vision, eye position, anterior segment, and fundus examinations. After that, a dilated optometry examination was performed in all students. For cycloplegia examination, two drops of cyclopentolate eye drops (1%) were administered to both eyes at a 5 min interval. After 20 min, if a pupillary light reflex was still present, a third drop was administered. The light reflex and pupil dilation were checked after an additional 15 min interval. Dilation and light reflex statuses were recorded 40–60 min after the first drop was administered. Cycloplegia was considered complete if the pupil dilated 6 mm or more and the light reflex was absent. Cycloplegic autorefraction was performed with an Topcon KR‐8900 autorefractor keratometer (OptoVision) for all the students. Refraction measurements, expressed as spherical equivalents (SE), were calculated as the algebraic sum of the spherical measurement and 0.5 times the cylindrical power. According to the maximum spherical equivalent (SE) value of the participant's lens, the students with myopia were divided into three groups: the mild myopia group (SE < 3.00 diopters), the moderate myopia group (SE 3.00–6.00 diopters), and the severe myopia group (SE > 6.00 diopters).

### 2.3 Questionnaires

Self‐Rating Anxiety Scale (SAS) and Self‐Rating Depression Scale (SDS).

Forms of the SAS and the SDS are widely used as simple diagnostic tools in both clinical and research settings (Li, Dai, & Zhang, 2017; Md, Mb, Mm, Mb, & Mb, 2019; Xie, Xie, Wang, Shu, & Dai, 2019). The SAS questionnaire is a 20‐item, self‐reported assessment that uses a four‐point Likert scale to rate the presence and severity of affective symptoms and somatic components of anxiety during the previous week (Zung, 1971). Fifteen of the items express negative experiences or symptoms (e.g., “I am afraid for no reason at all”), and five express positive experiences and are reverse‐scored (e.g., “I feel that everything is all right, and nothing bad will happen”). The SDS questionnaire is a 20‐item self‐reported assessment that uses a four‐point Likert scale to assess depression during the previous week (Zung, 1965). Ten of the items express negative experiences or symptoms (e.g., “I have crying spells or feel like it”), and ten express positive experiences and are reverse‐scored (e.g., “My life is pretty full”). Each question is scored on a scale of 4 (1, none or a little of the time; 2, some of the time; 3, much of the time; and 4, most of the time). The total score ranges from 20 to 80. For the SAS, the normal range is 20 to 44, the mild to moderate anxiety range is 45 to 59, the marked to severe anxiety range is 60 to 74, and the extreme anxiety range is 75 to 80 (Zung, 1971). For the SDS, the normal range is 20 to 49, the mildly depressed range is 50 to 59, the moderately depressed range is 60 to 69, and the severely depressed range is 70 and above (Zung, 1965). The reliability and validity of the SAS and SDS have been evaluated in several studies (Fountoulakis et al., 2001; Jegede, 1977; Olatunji, Deacon, Abramowitz, & Tolin, 2006), and the assessments have been standardized for the Chinese population (Jin & Zhang, 2017; Li, Jin, Qiao, Chen, & Gong, 2016).

### 2.4 Measurement

**Figure 1:**
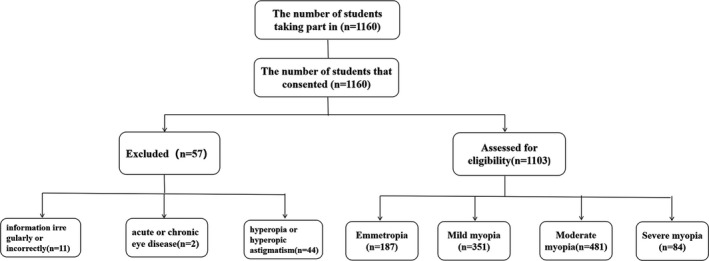
The flowchart for the participants in the study. First, a total of 1,160 students were consent and participated in the study. After completing an eye examination and questionnaire survey, 57 students were excluded, and the remaining 1,103 students were selected. These students were divided into emmetropia, mild myopia, moderate myopia, and severe myopia groups according to the results of the dilated optometry examination

The survey items covered demographics, general conditions, the SAS and the SDS. Demographic characteristics included gender and age. General conditions included physical health, history of medication, history of trauma, history of surgery, history of serious illness, satisfaction with vision, whether the participant wears glasses, habits associated with wearing glasses, learning stress, and learning time. After the investigator explained the procedures, all of the scales required the students to complete the SAS and SDS according to their psychological situation of the previous week. The students who had missing information, provided information irregularly or incorrectly, or could not complete the survey were excluded. A flowchart for participant recruitment is shown in Figure 1.

### 2.5 Statistical analysis

The data were analyzed using SPSS 23.0 statistical software (SPSS, Inc.), and processing methods for different data were adopted according to the characteristics of the data and the purpose of the research. In comparisons of scores between two groups, characteristics were evaluated for normality using the Shapiro–Wilk test; those with a normal distribution were compared using Student's *t* test, and those with a non‐normal distribution were compared using the Mann–Whitney test. When comparing more than two groups, the univariate analysis of variance with post hoc Bonferroni test or the ANOVA Kruskal–Wallis test by ranks was used. In comparisons of rates among groups, chi‐square tests were used. Spearman rank correlations were used to analyze the relationships among depression, anxiety, and degree of myopia. Values are presented as the mean ± *SD*. All reported *p*‐values are 2‐tailed, and statistical significance was defined at the α = 0.05 level.

## 3 RESULTS

### 3.1 Distribution of the survey population

A total of 1,160 people were surveyed, including 1,103 individuals who met the inclusion criteria and had complete survey data. The eligibility rate was 95.1%. Among the study participants, the rate of emmetropia (normal vision) was 16.9%, and the rate of myopia was 83.1%. The rate of myopia in boys was 82.3% and that in girls was 84.8%. The rates of myopia among 14‐, 15‐, 16‐, and 17‐year‐olds were 83.1%, 83.3%, 84.1%, and 82.7%, respectively. In addition, the rates of mild, moderate, and severe myopia were 38.3%, 52.5%, and 9.2%, respectively. The rates of myopia for different ages and genders were not significantly different (for age, χ^2^ = 0.256, *p* = .968, for gender, χ^2^ = 1.475, *p* = .225, Pearson's chi‐square).

### 3.2 Comparison of psychological health among students with emmetropia and myopia

To elucidate this relationship, we first compared the anxiety and depression rates of the students according to their age and gender. We found that there were no significant differences in the rates of anxiety and depression according to different ages and genders, indicating that the incidence of depression and anxiety among the first‐year high school students had little relation to their age or gender (Table 1). Second, we compared the anxiety and depression rates between the emmetropic group and the myopic groups. Our results showed that the anxiety rates of the myopic students were significantly higher than those of emmetropic students (χ^2^ = 11.463, *p* = .0001). However, although the myopic students had a higher depression rate than the emmetropic students, there was no significant difference between the groups (χ^2^ = 1.248, *p* = .264). Next, we divided anxiety and depression into mild, moderate, and severe levels according to the SDS and SAS scores. There were significant differences in the incidence rates of mild, moderate, and severe anxiety between the myopic and emmetropic students. However, there was a significant difference only in the rate of severe depression between the myopic and emmetropic students (Table 2). Finally, we analyzed the differences in the SDS and SAS scores between myopic and emmetropic students. We found that both SDS and SAS scores were significantly higher in the myopic students than in the emmetropic students (for the SDS, *t* = 5.678, *p* = .03; for the SAS, *t* = 7.035, *p* = .018; Student's *t* test, Table 3).

**Table 1:** Composition of anxiety and depression students in first‐year high school

	Total students (*n*)	Depressed students (*n*)	Depression rate (％)	Anxious students (*n*)	Anxiety rate (％)	Depression rate		Anxiety rate	
						χ^2^	*p*	χ^2^	*p*
Gender									
Male	585	205	34.7	222	37.9	0.028	.9	0.051	.852
Female	518	184	35.3	200	38.6				
Age (years)									
14	150	50	33.3	56	37.3	3.051	.384	0.641	.887
15	458	168	36.7	180	39.3				
16	347	107	30.8	133	38.3				
17	148	51	34.5	53	35.8				

Note

Compared with the boys, there were no significant differences in the rates of anxiety and depression in the girls. The differences in the anxiety rate and the depression rate among different ages were not significant (chi‐square test was used).

**Table 2:** Comparison of anxiety and depression rates among students with different degrees of myopia

Students	*N*	Anxiety rate (%)				Depression rate (%)			
		Overall	Mild	Moderate	Severe	Overall	Mild	Moderate	Severe
Emmetropia	187	31.0	18.2	11.2	1.6	29.4	15.5	13.4	0.5
Myopia	916	44.4	23.5	16.7	4.2	33.6	14.4	16.3	2.9
χ^2^		11.463	5.146	6.030	4.095	1.248	0.003	1.192	3.835
*p*		.001	.023	.014	.043	.264	.985	.275	.049

**Table 3:** SDS and SAS scores of the myopic students versus the emmetropic students

Group	SDS	SAS
Emmetropia	45.4 ± 10.9	43.9 ± 10.8
Myopia	52.3 ± 8.9	50.5 ± 9.8
*t*	5.678	7.035
*p*	.03	.018

Note

Compared with the emmetropic students, the myopic students’ SDS and SAS scores were significantly increased (Student's *t* test was used).

### 3.3 Comparison of the psychological health of students with different degrees of myopia

**Figure 2:**
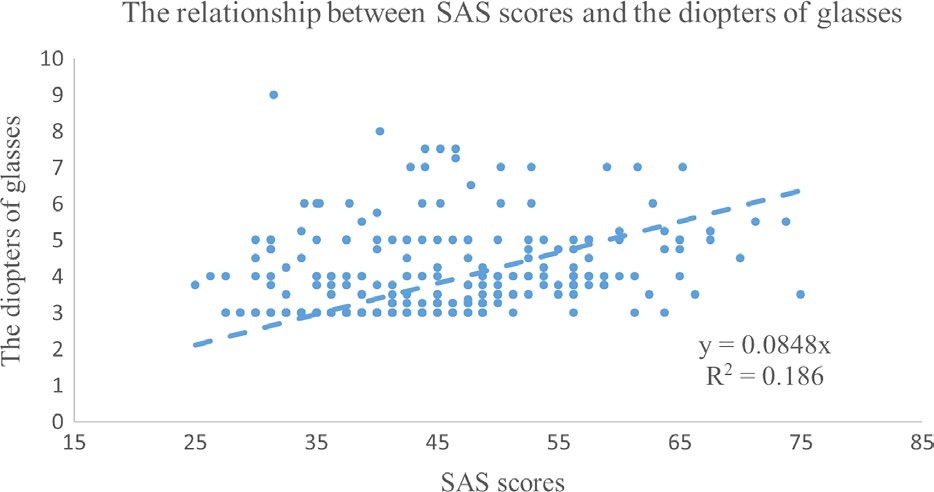
The relationship between the state of anxiety and the diopters of the participants’ glasses. There was a significant relationship between the SAS scores and the diopters of the glasses for all the students with myopia (*r* = 0.43, *p* = .045). The linear regression equation was y = 0.0848χ (*R* ^2^ = .186)

The SAS scores were significantly different among the severe, moderate, and mild myopia groups, while there were no significant differences among the three groups in the SDS scores (for the SAS, *F* = 10.342, *p* = .017; for the SDS, *F* = 1.008, *p* = .366, Univariate analysis of variance with the Kruskal–Wallis test, Table 4). This finding suggests the possibility of a closer relationship between anxiety and myopia than between depression and myopia. We also examined the relationships between the states of depression and anxiety and the diopters of the participants’ glasses. We found that the SAS scores were closely correlated with the diopters of the glasses for all the myopic students (*r* = 0.43, *p* = .045, Spearman rank correlation). However, the relationship between SDS scores and the diopters of glasses was not significant (*r* = 0.19, *p* = .325, Spearman rank correlation). We further explored the causal relationship between SAS scores and the diopters of glasses by linear regression. The linear regression equation was y = 0.0848χ (*R* ^2^ = .186), indicating that there was an approximately 18.6% possibility that when SAS scores increased by one point, the diopters of glasses would increase by 0.0848 diopters (Figure 2).

**Table 4:** SDS and SAS scores of the myopic students

	Myopia			*F*	*p*
	Mild	Moderate	Severe		
SDS	51.2 ± 10.3	52.9 ± 10.8	59.7 ± 10.5	1.008	.366
SAS	49.0 ± 10.2	52.0 ± 12.8	58.1 ± 15.1	10.362	.017

Note

The SAS scores were significantly different among the severe, moderate, and mild myopia groups, while there were no significant differences among the three groups in the SDS scores. (univariate analysis of variance with the Kruskal–Wallis test was used).

## 4 DISCUSSION

Although the incidences of both myopia and psychological illness have increased among adolescents in recent years, few studies are available on the relationship between them (Łazarczyk et al., 2016; Seitler, 2009; Ayaki et al., 2016). A review of the existing literature indicates that the current study is the first to our knowledge to examine the associations between adolescent anxiety and myopia and between adolescent depression and myopia. Recently, most related studies have focused on psychological health problems (e.g., anxiety or depression) among adolescents with high myopia (Ayaki et al., 2016; Yokoi et al., 2014), while we assessed the rates of anxiety and depression in groups with mild, moderate, and severe myopia and compared them with those of adolescents with emmetropia in the current study. Additionally, the relationships between the SDS and SAS scores and diopters of the students’ glasses were examined. This was also the first study to examine the relationship between the level of anxiety or/and depression and the degree of myopia in adolescent students.

Several hypotheses might explain why the incidence of anxiety and/or depression disorders is higher in people with myopia than in people without myopia. Seitler hypothesized that myopia is the result of a defense mechanism against tension that makes the extraocular muscles around the eyeball tighten, which directly causes refractive errors. Furthermore, tension causes discontinuity in the separation‐individuation process and myopic patients undergo separation anxiety that results in a sense of inability to cope with the world (Seitler, 2009). It is also thought that myopic students wearing glasses felt victimized due to overt bullying at school. Suffering in victims of bullying occurs due to stressful situations as well as dismissal to the margin of the group and a low social status among their peers (Juvonen et al., 2003). It has been found that feeling victimized in the early years of life may be related to psychosocial maladjustment and may cause an increase in anxiety, feelings of depression, and loneliness (Horwood et al., 2005; Hawker & Boulton, 2000; Lund, et al., 2009; Smokowski & Kopasz, 2005; Wolke, et al., 2000; Wolke, et al., 2001). Adolescents experience a period of rapid psychological and physiological development. They are susceptible to negative emotions, such as anxiety and depression, after a disaster or a change in their environment, and these emotions may continue to develop into affective disorders (Tang, Lu, & Xu, 2018). To study the psychological problems of adolescents with myopia, we examined students in their first year of high school, the age at which students can modulate the emotional and psychological fluctuations caused by academic stress. Although the students were all from one school grade, their ages ranged from 14 to 17 years. There were several reasons for this. First, in China, students enter primary schools at different ages. Most of them are 6 or 7 years old, but some students do not enter school until they are 8 years old. Second, some students did not perform well on their senior high school entrance examination (the examination for entrance into senior high school) and failed to start senior high school with their peers. After one year or multiple years, they passed the examination and then re‐entered senior high school at an older age. First‐year students have just experienced the pressure of entering a higher‐level school, thus showing their ability to resist stress and undergo an intense psychological experience (Zhang et al., 2018). They are able to recognize and evaluate themselves more accurately and provide a better representation of adolescent groups overall.

In this study, we found that the rates of depression among 14‐, 15‐, 16‐, and 17‐year‐olds were 33.3%, 36.7%, 30.8%, and 34.5%, respectively, and the corresponding rates of anxiety were 37.3%, 39.3%, 38.3%, and 35.8%, respectively, which were far higher than the 22% depression rate and 25% anxiety rate mentioned in Yokol's report (Yokoi et al., 2014). The differences between the two findings may be due to the following: first, the age of our participants ranged from 14 to 17 years, and most were 15 to 16 years of age, while Yokol focused on patients with myopia who were 40–50 years old; second, there were differences in living environments and the level of education. In addition, our research samples were more than 5 times the size of Yokol's, which makes our results highly reliable.

We found no significant difference in the depression rate between students with emmetropia and those with myopia, which was not consistent with the results of Wu et al. (2017), Ayaki et al. (2016), and Yokoi et al. (2014). Wu provided evidence that myopia was related to the presence of depressive symptoms among older adults, while Ayaki and Yokoi showed that high myopia significantly affected patients’ depression. As mentioned earlier, the participants in our study were adolescents, and depression rates among adolescents (those with either emmetropia or myopia) are higher than those among adults; thus, the difference between the two age groups might partly account for the difference in the outcomes. Next, we compared the depression rates between the emmetropic students and the students with different degrees of myopia (mild, moderate, and severe). We found that the depression rate among the students with high myopia was significantly higher than that among the students with emmetropia, but there was no significant difference in the depression rates between the students with mild and moderate myopia and the students with emmetropia. Because more than 90% of the first‐year high school students with myopia had mild to moderate myopia, the overall rate of depression was not significantly different between the emmetropic students and the myopic students, which was one of the reasons our results were inconsistent with those of Ayaki and Yokoi. However, we compared the SDS scores of the myopic and emmetropic students and found that the scores of the myopic students were significantly higher than those of the emmetropic students (*t* = 5.678, *p* = .03, Table 3). Moreover, the mean scores of the SDS were the highest in the severe myopia group, moderate in the moderate myopia group, and the lowest in the mild myopia group. These results suggested that myopia improved the SDS scores. However, the increases in SDS scores were limited, and they were not enough to increase the rate of depression. On the other hand, this finding also suggested that it was more accurate to evaluate depression by using SDS scores than by using rates because the former can detect more subtle changes in depressive states.

Our data showed that myopia was associated more closely with anxiety than depression in the first‐year high school students. In terms of the incidence rate, the rates of anxiety in students with myopia were significantly increased compared with emmetropic students, while the difference in the depression rate between myopic students and emmetropic students was not significant. In terms of the scores, the SAS scores of students with different degrees of myopia (including mild, moderate, and severe myopia) were significantly higher than those of the emmetropic students, while the SDS scores were only significantly higher among the students with high myopia. In terms of the correlation with myopia severity, the SAS scores, but not the SDS scores, were closely correlated with the diopters of glasses for all myopic students. Furthermore, we found that the correlation between the SAS scores and the diopters of glasses conformed to a linear regression and derived a linear regression equation that further demonstrated the close relationship between myopia and anxiety.

## 5 CONCLUSION

In summary, our study investigated the differences in the psychological health of first‐year high school students with myopia and those with emmetropia. Compared with the association between depression and myopia among adolescents, the association between anxiety and myopia was closer. Further research is needed to understand the link so that interventions can be developed.

## CONFLICT OF INTERESTS

No competing financial interests exist.

## AUTHOR CONTRIBUTION

Qiaoli Li, Jiezheng Yang, Tao Wang, and Shiqi Ling involved in conception and design. Tao Wang, Qiaoli Li, Ting Wang, Lei Zhong, Jiezheng Yang, and Ziqian Zhu involved in eye examination. Yan He, Lei Zhong, Ting Wang, Jiezheng Yang, and Ziqian Zhu involved in questionnaire survey: Qiaoli Li, Jiezheng Yang, Lei Zhong, Yan He, and Ting Wang contributed to collection and assembly of data. Qiaoli Li, Jiezheng Yang, Tao Wang, and Shiqi Ling involved in data analysis and interpretation. Qiaoli Li, Jiezheng Yang, Tao Wang, and Shiqi Ling involved in manuscript writing. All authors contributed to final approval of manuscript.

## Data Availability

The data that support the findings of this study are available from the corresponding author upon reasonable request.

## References

[brb31594-bib-0001] Andersen, J. R. , Aasprang, A. , Bergsholm, P. , Sletteskog, N. , Vage, V. , & Natvig, G. K. (2010). Anxiety and depression in association with morbid obesity: Changes with improved physical health after duodenal switch. Health and Quality of Life Outcomes, 8, 52 10.1186/1477-7525-8-52 20492663 PMC2881107

[brb31594-bib-0002] Ayaki, M. , Torii, H. , Tsubota, K. , & Negishi, K. (2016). Decreased sleep quality in high myopia children. Scientific Reports, 6, 33902 10.1038/srep33902 27650408 PMC5030671

[brb31594-bib-0003] Chandarana, P. C. , Eals, M. , Steingart, A. B. , Bellamy, N. , & Allen, S. (1987). The detection of psychiatric morbidity and associated factors in patients with rheumatoid arthritis. Canadian Journal of Psychiatry, 32(5), 356–361. 10.1177/070674378703200506 3651977

[brb31594-bib-0004] Derogatis, L. R. , Morrow, G. R. , Fetting, J. , Penman, D. , Piasetsky, S. , Schmale, A. M. , … Carnicke, C. J. (1983). The prevalence of psychiatric disorders among cancer patients. JAMA, 249(6), 751–757. 10.1001/jama.249.6.751 6823028

[brb31594-bib-0005] Ferrell, B. R. , & Hassey, D. K. (1997) Quality of life among long‐term cancer survivors. Oncology (Williston Park), 11(4), 565–568, 571, 572, 575–576. 9130276

[brb31594-bib-0006] Fountoulakis, K. N. , Lacovides, A. , Samolis, S. , Kleanthous, S. , Kaprinis, S. G. , St, K. G. , & Bech, P. (2001). Reliability, validity and psychometric properties of the Greek translation of the Zung Depression Rating Scale. BMC Psychiatry, 1, 6. 11806757 10.1186/1471-244X-1-6 PMC64635

[brb31594-bib-0007] Frasure‐Smith, N. , & Lesperance, F. (2008). Depression and anxiety as predictors of 2‐year cardiac events in patients with stable coronary artery disease. Archives of General Psychiatry, 65(1), 62–71. 10.1001/archgenpsychiatry.2007.4 18180430

[brb31594-bib-0008] Hawker, D. S. , & Boulton, M. J. (2000). Twenty years' research on peer victimization and psychosocial maladjustment: A meta‐analytic review of cross‐sectional studies. Journal of Child Psychology and Psychiatry, 41(4), 441–455. 10836674

[brb31594-bib-0009] Horwood, J. , Waylen, A. , Herrick, D. , Williams, C. , & Wolke, D. (2005). Common visual defects and peer victimization in children. Investigative Ophthalmology & Visual Science, 46(4), 1177–1181. 10.1167/iovs.04-0597 15790876

[brb31594-bib-0010] Jegede, R. O. (1977). Psychometric attributes of the self‐rating anxiety scale. Psychological Reports, 40(1), 303–306. 10.2466/pr0.1977.40.1.303 840986

[brb31594-bib-0011] Jin, T. , & Zhang, L. J. (2017). Introduction and application of self‐rating depression scales in China. Journal of Neuroscience and Mental Health, 17(5), 366–369. 10.3969/j.issn.1009-6574.2017.05.016

[brb31594-bib-0012] Jones, D. , & Luensmann, D. (2012). The prevalence and impact of high myopia. Eye & Contact Lens‐Science and Clinical Practice, 38(3), 188–196. 10.1097/ICL.0b013e31824ccbc3 22495679

[brb31594-bib-0013] Juvonen, J. , Graham, S. , & Schuster, M. A. (2003). Bullying among young adolescents: The strong, the weak, and the troubled. Pediatrics, 112(6 Pt 1), 1231–1237. 10.1542/peds.112.6.1231 14654590

[brb31594-bib-0014] Lazarczyk, J. B. , Urban, B. , Konarzewska, B. , Szulc, A. , Bakunowicz‐Lazarczyk, A. , Zmudzka, E. , Kowzan, U. , Waszkiewicz, N. , & Juszczyk‐Zajkowska, K. (2016) The differences in level of trait anxiety among girls and boys aged 13–17 years with myopia and emmetropia. BMC Ophthalmology, 16(1), 13–17. 10.1186/s12886-016-0382-2. 27842529 PMC5109705

[brb31594-bib-0015] Li, H. , Jin, D. , Qiao, F. , Chen, J. , & Gong, J. (2016). Relationship between the Self‐Rating Anxiety Scale score and the success rate of 64‐slice computed tomography coronary angiography. International Journal of Psychiatry in Medicine, 51(1), 47–55. 10.1177/0091217415621265 26681235

[brb31594-bib-0016] Li, Y. , Dai, W. , & Zhang, J. (2017). Anxiety, depression and quality of life in patients with a treated or untreated unruptured intracranial aneurysm. Journal of Clinical Neuroscience, 45, 223–226. 10.1016/j.jocn.2017.07.019 28778800

[brb31594-bib-0017] Lund, R. , Nielsen, K. K. , Hansen, D. H. , Kriegbaum, M. , Molbo, D. , Due, P. , & Christensen, U. (2009). Exposure to bullying at school and depression in adulthood: A study of Danish men born in 1953. The European Journal of Public Health, 19(1), 111–116. 10.1093/eurpub/ckn101 19008241

[brb31594-bib-0018] Md, T. L. , Mb, C. J. , Mm, Y. P. , Mb, W. Z. , & Mb, X. F. (2019). Correlation between premature ejaculation and psychological disorders in 270 Chinese outpatients. Psychiatry Research, 272, 69–72. 10.1016/j.psychres.2018.12.038 30579184

[brb31594-bib-0019] Morgan, I. G. , French, A. N. , Ashby, R. S. , Guo, X. , Ding, X. , He, M. , & Rose, K. A. (2018). The epidemics of myopia: Aetiology and prevention. Progress in Retinal and Eye Research, 62, 134–149. 10.1016/j.preteyeres.2017.09.004 28951126

[brb31594-bib-0020] Morgan, I. G. , Ohno‐Matsui, K. , & Saw, S. M. (2012). Myopia. The Lancet, 379(9827), 1739–1748. 10.1016/S0140-6736(12)60272-4 22559900

[brb31594-bib-0021] Olatunji, B. O. , Deacon, B. J. , Abramowitz, J. S. , & Tolin, D. F. (2006). Dimensionality of somatic complaints: Factor structure and psychometric properties of the Self‐Rating Anxiety Scale. Journal of Anxiety Disorders, 20(5), 543–561. 10.1016/j.janxdis.2005.08.002 16198532

[brb31594-bib-0022] Owsley, C. , McGwin, G. J. , Scilley, K. , Meek, G. C. , Seker, D. , & Dyer, A. (2007). Effect of refractive error correction on health‐related quality of life and depression in older nursing home residents. Archives of Ophthalmology, 125(11), 1471–1477. 10.1001/archopht.125.11.1471 17998508

[brb31594-bib-0023] Rose, K. E. , Tullo;, A. B. , & Flitcroft, D. I. (1998). Myopia. British Journal of Ophthalmology, 82(10), 1220–1221. 10.1136/bjo.82.10.1220a PMC1722396 9924318

[brb31594-bib-0024] Seitler, B. N. (2009). Separation‐individuation issues and castration anxiety: Their curious influence on the epigenesis of myopia. American Journal of Psychoanalysis, 69(3), 221–237. 10.1057/ajp.2009.14 19794421

[brb31594-bib-0025] Skarstein, J. , Aass, N. , Fossa, S. D. , Skovlund, E. , & Dahl, A. A. (2000). Anxiety and depression in cancer patients: Relation between the hospital anxiety and depression scale and the european organization for research and treatment of cancer core quality of life questionnaire. Journal of Psychosomatic Research, 49(1), 27–34. 10.1016/s0022-3999(00)00080-5 11053601

[brb31594-bib-0026] Smokowski, P. R. , & Kopasz, K. H. (2005). Bullying in school: An overview of types, effects, family characteristics, and intervention strategies. Children & Schools, 27(2), 101–110. 10.1093/cs/27.2.101

[brb31594-bib-0027] Stapinski, L. A. , Bowes, L. , Wolke, D. , Pearson, R. M. , Mahedy, L. , Button, K. S. , … Araya, R. (2014). Peer victimization during adolescence and risk for anxiety disorders in adulthood: A prospective cohort study. Depression and Anxiety, 31(7), 574–582. 10.1002/da.22270 24788688 PMC4190687

[brb31594-bib-0028] Tang, W. , Lu, Y. , & Xu, J. (2018). Post‐traumatic stress disorder, anxiety and depression symptoms among adolescent earthquake victims: Comorbidity and associated sleep‐disturbing factors. Social Psychiatry and Psychiatric Epidemiology, 53(11), 1241–1251. 10.1007/s00127-018-1576-0 30109368

[brb31594-bib-0029] Vitale, S. , Sperduto, R. D. , & Ferris, F. R. (2009). Increased prevalence of myopia in the United States between 1971–1972 and 1999–2004. Archives of Ophthalmology, 127(12), 1632–1639. 10.1001/archophthalmol.2009.303 20008719

[brb31594-bib-0030] Wolke, D. , Woods, S. , Bloomfield, L. , & Karstadt, L. (2000). The association between direct and relational bullying and behaviour problems among primary school children. Journal of Child Psychology and Psychiatry, 41(8), 989–1002. 11099116

[brb31594-bib-0031] Wolke, D. , Woods, S. , Stanford, K. , & Schulz, H. (2001). Bullying and victimization of primary school children in England and Germany: Prevalence and school factors. British Journal of Psychology, 92(Pt 4), 673–696. 10.1348/000712601162419 11762868

[brb31594-bib-0032] Wu, N. , Kong, X. , Gao, J. , & Sun, X. (2019). Vision‐related quality of life in glaucoma patients and its correlations with psychological disturbances and visual function indices. Journal of Glaucoma, 28(3), 207–215. 10.1097/IJG.0000000000001178 30624385

[brb31594-bib-0033] Wu, Y. , Ma, Q. , Sun, H. P. , Xu, Y. , Niu, M. E. , & Pan, C. W. (2017). Myopia and depressive symptoms among older Chinese adults. PLoS ONE, 12(5), e177613 10.1371/journal.pone.0177613 PMC5428930 28498851

[brb31594-bib-0034] Xiang, F. , He, M. , Zeng, Y. , Mai, J. , Rose, K. A. , & Morgan, I. G. (2013). Increases in the prevalence of reduced visual acuity and myopia in Chinese children in Guangzhou over the past 20 years. Eye (Lond), 27(12), 1353–1358. 10.1038/eye.2013.194 24008929 PMC3869515

[brb31594-bib-0035] Xie, Y. H. , Xie, H. T. , Wang, T. S. , Shu, Y. P. , & Dai, X. L. (2019). Perioperative holistic care more significantly reduces levels of anxiety and depression of pituitary tumor patients versus conventional care. Medicine (Baltimore), 98(7), e14411 10.1097/MD.0000000000014411 30762744 PMC6407947

[brb31594-bib-0036] Yokoi, T. , Moriyama, M. , Hayashi, K. , Shimada, N. , Tomita, M. , Yamamoto, N. , … Ohno‐Matsui, K. (2014). Predictive factors for comorbid psychiatric disorders and their impact on vision‐related quality of life in patients with high myopia. International Ophthalmology, 34(2), 171–183. 10.1007/s10792-013-9805-8 23783655

[brb31594-bib-0037] Zhang, D. , Cui, Y. , Zhou, Y. , Cai, M. , & Liu, H. (2018). The role of school adaptation and self‐concept in influencing Chinese high school students' growth in math achievement. Frontiers in Psychology, 29, 2356 10.3389/fpsyg.2018.02356 PMC6282037 30555381

[brb31594-bib-0038] Zung, W. W. (1965). A self‐rating depression scale. Archives of General Psychiatry, 12, 63–70. 14221692 10.1001/archpsyc.1965.01720310065008

[brb31594-bib-0039] Zung, W. W. (1971). A rating instrument for anxiety disorders. Psychosomatics, 12(6), 371–379. 10.1016/S0033-3182(71)71479-0 5172928

